# Hospital Economies
*A paper read at the Twelfth Conference of the British Hospitals Association, May 26, 1922.


**Published:** 1922-08

**Authors:** Frank G. Hazell

**Affiliations:** (General Superintendent and Secretary, Manchester Royal Infirmary)


### HOSPITAL ECONOMIES.*

By FRANK G. HAZELL (General Superintendent and Secretary, Manchester Royal Infirmary).

THE subject set down for consideration having for its root " House—Law," it will be well to consider the site of a hospital. So far as speed is concerned, in these days of motor transport, it matters little, from the point of view of saving life, whether a hospital is situated in the centre of a town or city, or a mile distant. But from the point of view of economy it makes a vast difference. Out of sight is out of mind. The daily reminder of its presence, its work, and all that it stands for is at once an appeal and an inspiration, the economic value of which one cannot afford to ignore.

What opportunities for economy are missed in the plan and construction of our hospitals, even the most modern! So often one finds that a hospital was planned literally for its first requirement, that any extension proves too expensive and any alteration will upset the balance of the whole. The administration block invariably proves too small immediately there is any demand for the extension of the wards or the establishment of new departments. Unlike Nature, we do not provide " brain room " for the accommodation of the new needs of a growing body.

Under the Uniform System of hospital accounts, the first nine headings represent from one-half to two-thirds of the ordinary income. A comparison of income received under these headings by county hospitals, sub-county hospitals, and cottage hospitals, each in its separate class, is eloquent of initiative in some instances, and the extraordinary lack of it in others. In many instances insufficient income is directly attributable to want of publicity. A hospital has all too few opportunities of advertising the good work it is doing. The board of management is voluntary, so is the medical staff, and until comparatively recently the nurses were also. Modesty and professional etiquette combine to prevent any real use being made of advertisement in connection with the wonderful work which is proceeding within its walls. Consequently a constant supply of rich material for propaganda is cut off at its source and wasted, material that would stir the imagination of beneficently disposed but otherwise unimaginative persons to the financial benefit of the hospital. Lack of imagination and initiative and reluctance to advertise are the first reasons for the want of sufficient monetary support. The voluntary system of hospital management is the most efficient, the most economical and the most humane; and, as has been rightly said, it must be maintained. But it cannot afford to retain its present attitude towards publicity.

King Edward's Hospital Fund is applicable to London only, and benefits some 108 hospitals within a 10-mile radius of Charing Cross, and 62 convalescent homes and sanatoria. London has derived immense benefit from its fund for 25 years past. It is a noticeable feature of the London Fund, judging by the announcements which appear from time to time in the newspaper Press, that a large proportion of its income is derived from those who have amassed their fortunes in the provinces and colonies, but however much they desire to benefit the hospitals generally, there is only this one central fund. A " King George's Hospital Fund for Provincial Hospitals " is wanted urgently, and it is high time such a fund was established for the benefit of the 650 provincial hospitals. The reception and disposal of income arising from large benefactions and bequests need not be its sole function. Co-ordination is what we lack most. Its value is recognised by the Visiting Committee of the King Edward's Hospital Fund for London. It was recognised by the late Sir Henry Burdett. And there is the record of Sir Napier Burnett's valuable service of co-ordination during the war. It would be a function of this new Fund to co-ordinate the work of the provincial hospitals.

The application of the principle of inspection of all voluntary hospitals throughout the provinces, followed by a report by official visitors of proved capacity, especially if the visit is supported by the prospect of a share in a grant if the hospital earns a favourable report, would have an extraordinarily enlivening effect. But such visits and reports, to be of real benefit, must be constructive. The visitors must be in a position to show by chart, or statement, what other hospitals are doing. They must be furnished with facts by competent clerks able to tabulate information and to supply them with any details they require for the purpose of their visit, and to send subsequently to the hospital visited helpful information concerning other hospitals, on points referred to in the visitors' report.

First, let the hospitals benefit by constructive criticism and friendly rivalry as between them and their departments. Then, let participation in a grant be regarded as recognition by experts of the efficient conduct of every department. The actual proportion of the grant should be based, not on overdraft, not on estimated income, but upon work done. With the exception of the provision of, say, half the cost of extensions proved to be a necessity, it is questionable whether the time for a yearly grant from the Government has arrived, because it has yet to be proved that every avenue of possible income has been explored, or that finality has been reached in the matter of economy. Helpful, constructive criticism by men who know the work would prove of the greatest value. A request that the visitors' reports be published could not be denied, and public opinion could be trusted to see to the rest. No voluntary hospital can afford to maintain an attitude of indifference with regard to the management of other hospitals. Every hospital in the country has something to gain by skilled criticism and comparison of its management with that of other hospitals.

Hospital Sunday and Saturday Funds are not established universally. Where they are established the amount collected varies so extraordinarily as to

* A paper read at the Twelfth Conference of the British Hospitals Association, May 26, 1922.

bear no relationship to the population, either church-going or working class. Nor is comparison possible in any case with the sum raised in another town of similar size. The composition of the Committee and the choice of officials is largely responsible for failure, because in all walks of life the social status of the applicant carries influence, and this is a class of philanthropic work in which influential people do not interest themselves as largely as they might. Hospital Sunday affords an excellent opportunity for propaganda. In place of the bare announcement, *inter alia*, " the collection at this service will be given to the hospitals," the fact should be well advertised beforehand that Dr. or Mr. So-and-so will give an address on Hospital Sunday. Such addresses would be academic, historic, appealing, as suited the mind of the speaker, resulting in an intelligent interest being taken in the work of the hospitals by a widely increased circle.

The annual revision of investments is a matter which might very profitably be recommended.

The subject of patients' payments has received wide publicity by reason of the introduction here and there of a fixed charge to cover the cost of maintenance. The system has been hailed as the remedy for deficient incomes everywhere, and so the fact has been lost sight of that a hospital situated in the provinces cannot be compared with one of the London hospitals in this respect. While patients have another hospital to which they can go, a system of payment may appear to succeed ; but when the system has been adopted by all the general and special hospitals, one of two things must happen : Either (1) it must fail for the reason that from 40 to 50 per cent. of in-patients are unable to contribute anything appreciable towards the cost of their maintenance, or (2) that percentage (40 to 50) must go to the workhouse hospitals. This apparently high percentage is accounted for by the fact that : (1) Male medical patients have usually been ill, and consequently out of work, for some while before admission ; (2) female medical patients have usually been ill and consequently an expense for some while prior to admission ; (3) male surgical patients have to rely on compensation, or sick pay from Friendly or Approved Societies, for the support of their families ; (4) if the patient is the mother of a family, a foster-mother has to be employed, and the household expenses are thereby increased. The bulk of contributions comes from the male surgical patients. In a report issued by one of the London hospitals after this scheme had been worked for three months, it was stated that : " 10 per cent. only of those applying for admission were unable to pay the charge of a guinea each week." It is obvious that those who were sensitive of proclaiming their poverty, sought admission elsewhere.

Voluntary hospitals depend on goodwill for half their income. If they make the payment of a fixed charge a condition of admission they stand to lose the whole of their voluntary contributions by patients, the bulk of their box collections in the hospital itself, and many of their annual subscriptions, donations, and workshop collections. If they lose their goodwill, wherein lies the economy ? In any case a fixed charge is an empty threat, because payment cannot be enforced. The voluntary hospitals were established, and are maintained, for the benefit of the *sick poor*, not as a species of cheap nursing home, for which there is so large a demand at the present time. The people accept the position that if you are insane, or are suffering from fever or consumption, you are " removed," you have no option. But to seek admission to the workhouse infirmary of your own free will is quite another matter. While any choice remains, the workhouse infirmaries will have empty beds and the voluntary hospitals will have waiting lists. This factor increases in importance as the population of the city or town diminishes, and facilities for discussing your neighbour's affairs increase.

Until recently the National Health Insurance Act contained a clause whereby a person having no dependents could assign his or her benefits to the hospital, and the hospitals derived a considerable sum from this source. The Act, as revised, provides that the Approved Society must pay sickness benefit to the insured person. Payment is delayed, with the result that in the meantime the patient's gratitude has waned, and the hospital does not receive anything. It is a clause which might be reinstated with great advantage. To ignore this loss is extravagant.

Under the uniform system of hospital accounts, the cost of maintenance is distributed approximately as follows :—

| | Per cent. |
|---|---|
| A.—Provisions, Domestic and Miscellaneous | 46 |
| B.—Surgery and Dispensary ... ... | 8¼ |
| C.—Establishment ... ... ... ... | 6¼ |
| Salaries ... ... ... ... ... | 39½ |

The control of this expenditure devolves, according to the size of the hospital, upon :—

A.—The steward.
B.—The pharmacist.
C.—The clerk of works.

A.—There are very few hospitals in the country where the services of a trained buyer are recognised as necessary, and yet a hospital of 300 beds will spend £30,000 a year under these three headings, Provisions, Domestic and Miscellaneous. Consequently the purchase of the majority of the component items is made by contracts varying in length of term.

B.—The pharmacist in many instances buys by six months' contract, or by quarterly or monthly price-list. It is the exception to find him at liberty to buy in the open market.

C.—The employment of a clerk of works in the supervision of the maintenance of the fabric is a sound investment.

A. & B.—Purchase by contract is, in the main, an expensive method. Buying to meet requirement involves the careful watching of the markets, and requires freedom to apply the courage of your convictions ; it also entails considerable additional anxiety upon the responsible officials, but it is the means of effecting very considerable economies, Responsibility, though centralised in the secretary, should be decentralised to subordinate responsible heads such as a finance secretary, a steward, a pharmacist, and a clerk of works. With the immediate responsibility for the conduct of all the details of these four departments lifted from his shoulders, the secretary's whole time would still be fully occupied, but with far greater advantage economically, and therefore financially, to the hospital he is serving.

There are 650 voluntary hospitals in England and Wales, excluding London; of these, 450 have less than 100 beds each. Advocates of co-operative buying claim that it would assist the small hospital. It has been tried, and has proved a failure. To obtain the bulk of your purchases outside the town in which your small hospital is situated tends to alienate sympathy, if not to antagonise the tradesmen, who, as a general rule, like to have your account either on sentimental or business grounds, and will quote you favourable terms to retain it. The expense incurred in establishing and conducting a central store, plus the cost of delivery, must absorb any margin of profit secured by purchasing in bulk. There is also the possibility to consider of wholesale and retail traders generally resenting such an action on the part of the hospitals, and they have it in their power to retaliate. Further, it must be admitted that the likelihood of every small hospital coming into the scheme is extremely remote. The hypothetical economy to be effected by the adoption of such a scheme is so small as to make it not worth while. Co-operative buying would kill initiative in the very officials in whom it should be cultivated. Responsibility for slackness and the appalling wastefulness on the part of the consumers of to-day is largely traceable to the method of "indenting," acquired during the war.

### Conclusion.

The question of site and building is one which will arise very rarely, but is worth bearing in mind. By clever publicity the Norfolk and Norwich Hospital increased its ordinary income during 1921 from £36,000 to £54,000. Hospital Sunday and Hospital Saturday Funds offer wider opportunities than are at present utilised. "Patients' payments" is a misnomer, as in the great majority of hospitals they are contributions made with a free will and are in no sense a payment in exchange for something received. If we are to retain the spirit of voluntaryism which is the soul of our hospitals, not only must the services of the board of management and the medical staff remain voluntary, but recognition by the patients must also be voluntary. We have nothing to learn from Continental methods but what to avoid in this respect. The introduction of co-operative buying may be left to create its own demand.

There are instances of economy I have not named, such as the value of exerting silent pressure by the submission to the medical and nursing staff of graphs, quantities and costs, showing a comparison of the working of their several departments; departmental costing and its comparison with that of similar departments in other hospitals; amalgamation with small special hospitals; and many others.

There remains the question of establishing a Central Fund for the 650 provincial hospitals. The disposal of the National Relief Fund, the British Red Cross Fund, and the Government grant to the hospitals with the largest deficit, by putting a premium on sloth and extravagance, does not meet the difficulty, but, in conjunction with the findings of the Voluntary Hospitals Commission, serves to draw attention to a pressing need which local Voluntary Hospitals Committees will not in the future be able to fulfil. A committee which has no fund to disburse cannot exert any economic pressure. Scores of cases occur in the course of each year in which the disposal of the residue of an estate is left in the absolute discretion of the executors. If such a fund as has been indicated were inaugurated, it would be supported. But it could not be expected to be conducted on such economical lines as that for London, where, with the exception of a few sanatoria, all of the 170 hospitals and convalescent homes are situated within easy reach of Charing Cross, with the result that the average expenses do not exceed 25s. per cent., or 3¼d. in the £. King Edward's Hospital Fund for London has been established 25 years. It has stood the test of time, and it has succeeded, because of confidence in its personnel, because of its convenience, because of its economical management, because the control it exercises is a live force. A King George's Hospital Fund for the Provinces would prove just as attractive, and, therefore, successful; just as convenient, and, considering the vastly greater number of hospitals it would influence, its power for good would be increased, and in the exercise of that power it would become a vitalising influence, not only as affects expenditure, and the control of it, but income also, in every provincial hospital.

### THE FUTURE OF THE HOSPITALS.

The Conference held at the Ministry of Health on July 18 and 19, at which Lord Onslow, Chairman of the Voluntary Hospitals Commission, presided, marks the end of a chapter in the history of voluntary hospitals. Members of the Committees that have been formed during the past few months attended. On the first day there was considerable discussion on the question of the payment of administrative expenses. On the second wider issues were raised.

The whole future of the voluntary hospitals and the directions in which they can be assisted by the Committees were discussed in their various aspects. Among the questions raised were the contributions from approved societies, on which Sir Walter Kinnear. Controller of the Insurance Department, made a statement; the relation between poor law infirmaries and voluntary hospitals; the advantages to be gained from a uniform system of accounting; and the possibilities of extending small regular contributions from industry.

As this Review went to press before the Conference was concluded, it was impossible to publish a full account of the proceedings. Arrangements have, however, been made to give an authoritative account of the decisions arrived at in the September issue. It is satisfactory to know from Lord Onslow that the present Government intends to do all possible to maintain voluntary hospitals.

